# A First Principles Approach to Subjective Experience

**DOI:** 10.3389/fnsys.2022.756224

**Published:** 2022-02-16

**Authors:** Brian Key, Oressia Zalucki, Deborah J. Brown

**Affiliations:** ^1^School of Biomedical Sciences, University of Queensland, Brisbane, QLD, Australia; ^2^School of Historical and Philosophical Inquiry, University of Queensland, Brisbane, QLD, Australia

**Keywords:** sentience, awareness, phenomenal consciousness, feelings, qualia

## Abstract

Understanding the neural bases of subjective experience remains one of the great challenges of the natural sciences. Higher-order theories of consciousness are typically defended by assessments of neural activity in higher cortical regions during perception, often with disregard to the nature of the neural computations that these regions execute. We have sought to refocus the problem toward identification of those neural computations that are necessary for subjective experience with the goal of defining the sorts of neural architectures that can perform these operations. This approach removes reliance on behaviour and brain homologies for appraising whether non-human animals have the potential to subjectively experience sensory stimuli. Using two basic principles—first, subjective experience is dependent on complex processing executing specific neural functions and second, the structure-determines-function principle—we have reasoned that subjective experience requires a neural architecture consisting of stacked forward models that predict the output of neural processing from inputs. Given that forward models are dependent on appropriately connected processing modules that generate prediction, error detection and feedback control, we define a minimal neural architecture that is necessary (but not sufficient) for subjective experience. We refer to this framework as the *hierarchical forward models algorithm*. Accordingly, we postulate that any animal lacking this neural architecture will be incapable of subjective experience.

## Introduction

The subjective experience of sensory stimuli is variously referred to as conscious awareness, subjective awareness, inner awareness, phenomenal consciousness, qualia, and feelings. A commonly accepted description of subjective experience is that it is the “what it is like” experience of internal neural processing that typically arises from a sensory stimulus. There are two dimensions to the experience—first, there is the experience of something rather than nothing and then, second, there is the nature of the content of the experience (e.g., form and location in the case of the visual system). Although there are many different theories of subjective consciousness, we are chiefly interested here in theories that derive from the broad field of neuroscience. Those theories that fall outside of this category include physical theories such as the field theory (consciousness as a property of quantum-like processes; John, 2001), the quantum theory (consciousness is a fundamental property of matter; Gao, 2008), the resonance theory (involves resonating or vibrating structures that enable phase transitions; Hunt and Schooler, 2019), the electromagnetic field theory (Pockett, 2002) as well as philosophical theories such as phenomenal externalism (consciousness is not in the brain but in the external world; Pautz, 2014) and dualism (consciousness is a fundamental property that is non-reducible to physical properties; Chalmers, 1995). In contrast to these aforementioned theories, neuroscientific theories of consciousness are based on the ability of nervous systems to process neural activity and execute neuronal operations on information-bearing states in order to perform functions, solve problems and achieve goals (Hopfield, 1994; Koch and Laurent, 1999). While we favour neuroscientific theories of consciousness here, none have, as yet revealed the nature of the neural computations that generate subjective experience. Explaining how a physical system such as a brain can generate subjective experience remains a major challenge.

Philosophically, the form of reasoning we use here is the classical one of arguing from first principles. In Book I of his Physics, Aristotle seeks to understand nature through knowledge of basic or primary causes, i.e., “first principles” (Irwin, 1989). In this paper, we adopt a “first principles” approach to better understand the neural basis of subjective experience. We begin in section “Subjective Experience Is Contingent on Neural Processes” by simply defending the basic principle that subjective experience is contingent on specific neural processes. This is not a controversial premise within the context of neuroscientific theories of consciousness, and it is foundational with respect to subsequently clarifying those processes that are necessary for subjective experience. In section “What Some Theories of Consciousness Do Not Reveal About Subjective Experience,” we provide an overview of why some extant and popular theories of consciousness that are wanting with respect to understanding the neural basis of subjective experience. In section “Applying First Principles to Understand the Neural Basis of Subjective Experience,” we introduce some key neural functions that support subjective experience. In section “The Neural Architecture Necessary for Subjective Experience,” we discuss the neural architecture that underpins these functions and then, in section “Conclusion,” finish with some concluding insights. By adopting a bottom-up strategy based on first principles, we propose that it may be possible to characterise both necessary and sufficient conditions for subjective experience, but our focus here is only on necessary conditions.

## Subjective Experience Is Contingent on Neural Processes

Our approach begins with the basic principle that subjective experience is dependent on neural processing involving the execution of specific functions rather than merely being a result of the firing of neurons. For example, others have argued that neural firing of C-type peripheral sensory neurons just is pain (Putnam, 1960)—an idea that has strongly influenced philosophical mind-brain debates (Puccetti, 1977; Levin, 2005; Montero and Brown, 2018; Polák and Marvan, 2018; Van den Hombergh, 2020). Rather than being pain, C-type firing may be just background noise without eliciting sensation (Schäfers and Cain, 2004; Ermentrout et al., 2008). Alternatively, given that C-fibres are polymodal (Perl, 2007), firing of these neurons may instead represent either nociception or innocuous heat/cold/mechanical sensations. Aside from lacking mechanistic explanatory power, such claims of type-type identity are focussed at the wrong level of abstraction just as claiming that water is an oxide would be. A more promising view is that subjective consciousness is not neural activity *per se* but rather a specific type of neural process (Place, 1956; Smart, 1959; Polger, 2011; Polák and Marvan, 2018). How, though, could a neural process be the same thing as a subjective experience? An analogy can be found in the arithmetic operation of summing two numerals. The process of summing is addition and addition just is the process of summing. Addition is not something “over and above” the computational process itself. Accepting type-type identity between subjective experience and certain kinds of neural processes would resolve the problem facing dualist or epiphenomenalist accounts that a non-materialistic subjective experience cannot have causative power because if subjective experience is just a physical process, it can be causative. Nonetheless, the challenge of identifying those neural processes that are subjective experiences would remain. We are a long way from being able to specify necessary and sufficient conditions for subjective experience.

Perhaps we can, however, say something about what kinds of neural processes are a necessary condition for subjective experience? To do so we must face an issue raised by the assumption that mental states are multiply realisable (Polger, 2011; Elgin, 2020). The idea is that if different neural structures can generate the same functional process in different brains, then it is impossible for any specific type of neural structure to be necessary for subjective experience. We have argued previously that multiple realisation need not apply at all levels of abstraction (Brown and Key, 2021a), and hence constitutes an unwarranted assumption in arguments against identity theory. Returning to the analogy of mathematics, it is obvious that arithmetic operations can be multiply realised at some level of description. For instance, the numbers 23 and 56 can be added in three-steps as either 20 + 50 and 3 + 6 and then 70 + 9. Alternatively, 23 + 56 can also be added as 2 + 5 = 7 for the first digit and as 3 + 6 = 9 for the second digit and then written as 79 without a third addition step. But while such an arithmetic operation can be multiply realised through application of different algorithms, they share a common structural feature which can be represented algebraically. Take the equation 2 + 5 = 7. Abstracting from the actual arguments and values, this can be represented as “x + y = z.” Of course, for any n-place formula, in this case, a two-place one, alternative n + 1-place formulae are possible—e.g., “x + y + r = z” or “x + y + r + s = z”—but the two-place formulation is a *minimal structural condition* for the realisation of the addition function. We are interested in identifying a minimal structural condition for the possibility of subjective experience. Despite variability in the numbers of neuronal elements at the micro level or differences in morphological expression of those processes at the macro level, we propose it is possible—at a certain level of abstraction—to identify necessary minimal architectures performing the function of subjective experience. If such structures could be identified, they could be used to support the inference that creatures lacking such architectures would not be capable of subjective experience. Abstract characterisation of a minimal neural architecture has been used to explain the possibility of left-right locomotion despite variation in the number of neuronal elements (Brown and Key, 2021a), suggesting that the same strategy might work for subjective experience as well. The problem would then become the empirical one of identifying the correct level of abstraction and the minimal structural condition (we return to this matter in section “Applying First Principles to Understand the Neural Basis of Subjective Experience”).

Some find type-type identity theory objectionable because it seems to imply that by accepting that subjective experience is a physical process, one is denying the qualitative nature or “feeling” of the experience itself. However, identity theory does not deny that subjective experience feels like something—it merely claims that that feeling is a physical brain process rather than something non-physical. The realisation that the properties of water can be explained as those of H_2_O is rather uncontroversial simply because both the properties and chemical composition are physical entities. But there supposedly arises an “explanatory gap” (Levine, 1983; Block and Stalnaker, 1999) when attempting to explain subjective qualities as the properties of neural states. Some, such as Papineau (2020), argue that the explanatory gap simply dissolves once subjective experience is accepted as nothing more than a physical process, but while we may understand the chemical properties of water as those of H_2_O, it seems impossible to imagine its taste based on knowing its chemical composition (unless one has already tasted it). And even once tasted, we are none the wiser about why it tasted like something rather than nothing. It is this mystery that sustains the explanatory gap.

Whether a neural process *causes* subjective experience or *is* subjective experience remains hotly debated (Polák and Marvan, 2018). Nonetheless, there is ample evidence from experimental manipulations and disease pathologies to support the basic principle that specific neural processes are necessary for subjective experience (Key and Brown, 2018). In the next section, we discuss how some of the leading neuroscientific theories of consciousness fail to adequately address the nature of these neural processes in subjective experience.

## What Some Theories of Consciousness Do *Not* Reveal About Subjective Experience

The *Global Neuronal Workspace* theory is considered a type of first-order theory (i.e., involving neural processes directly associated with sensory properties of stimuli). Such theories propose that consciousness arises when the contents of sensory processing are broadcast widely (and rapidly, like a sudden and intense ignition spark) across a workspace in the cerebral cortex that includes the prefrontal, temporal, and parietal cortices (Brown et al., 2019; Mashour et al., 2020). Panagiotaropoulos et al. (2020, p.180) state that “the contents of the workspace is (*sic*) what we subjectively experience as a conscious feeling or experience.” The *Global Neuronal Workspace* theory is supported by a wealth of experimental data obtained from investigations of conscious vision (Dehaene et al., 2017; Mashour et al., 2020). Typically, these visual studies compare brain activity between unseen and seen stimuli, using techniques such as masking, attentional blink, inattentional blindness, binocular rivalry, and binocular flash suppression (Dehaene et al., 2001; Sergent et al., 2005; Panagiotaropoulos et al., 2012; Pitts et al., 2012; Frässle et al., 2014). For example, in visual masking, background activity during unseen stimuli is subtracted from that recorded during seen stimuli to reveal brain regions of interest. For instance, a visual stimulus (such as a word) is briefly flashed for tens of milliseconds (e.g., ∼30 ms) and then this is followed immediately by a second conflicting and noisy stimulus (e.g., for ∼70 ms) (Dehaene et al., 2001). In this scenario the subject reports only perceiving the second stimulus. If the presentation of the second stimulus is sufficiently delayed, then the subject also consciously perceives the first stimulus.

When interpreting the significance of these experimental paradigms, one needs to distinguish between the contents of visual processing and the subjective experience of those contents. In both masking and non-masking, the subject is always consciously perceiving visual stimuli. That is, there is a visual experience of something rather than nothing. What is clearly different between the two conditions is the content of that experience. During masking, subjects perceive the mask whereas during control conditions, both mask and the initial target stimulus are perceived. When the neural signals are subtracted from each other, the activity associated with perceiving something rather than nothing is removed, leaving predominantly only that activity correlating with the contents of the visual experience (e.g., a word). What is lost in this experimental paradigm is the brain activity of interest—i.e., the activity associated with the subjective experience of something (no matter its content). Similar contrastive methods are used in the other techniques mentioned above.

Using a different strategy in which a visual stimulus is presented at threshold, it is possible to compare neural activity of a constant stimulus when it is either seen or unseen. Employing transcranial magnetic stimulation to generate simple visual precepts (flashes of light) removes the need for an external visual stimulus. Then, by systematically adjusting the magnetic stimulation levels, conscious phosphenes can be produced in approximately 50% of recordings (Taylor et al., 2010). This enables neural activity to be compared when phosphenes are either seen or not seen. This paradigm dispenses with the need for masks and by using a simple percept removes neural processing associated with discrimination and object recognition. However, a constant visible cue continues to be used as a fixation point, and because the display screen does not fill the entire visual field of the subject, there are other contaminating visual inputs. By subtracting the neural activity of unseen from seen phosphenes, the neural activity responsible for subjective experience is again removed, leaving the activity associated with visual content (i.e., phosphenes) and not subjective experience.

While subtractive approaches provide insight into the neural regions associated with conscious recognition or conscious content, they do not expose the neural basis of subjective experience itself. We note that others also find the contrastive approach of seen and unseen stimuli to be wanting with respect to understanding the neural basis of subjective experience, but for different reasons (Lepauvre and Melloni, 2021). Dehaene and others have also confessed that the Global Neuronal Workspace theory only seems to account for the conscious contents of visual experience and not the visual subjective experience itself (Graziano et al., 2020; Panagiotaropoulos et al., 2020; Rosenthal, 2020). So, we are left with a theory that does not speak to why the sudden ignition of global broadcasting should feel like something rather than nothing. Rosenthal had earlier proposed that global broadcasting could perhaps instantiate consciousness through downstream higher-order awareness processes (Rosenthal, 2012). Indeed, it has recently been suggested that subjective experience could arise from high-order self-monitoring processes occurring after global broadcasting (Graziano et al., 2020; Panagiotaropoulos et al., 2020). The nature of that self-monitoring is debatable. Dehaene et al. (2017) have suggested it could be meta-cognition, whereas Rosenthal (see below) considers that subjective experience is mediated instead by an intermediary level of higher-order awareness occurring before meta-cognition.

*Higher order theories* of consciousness are varied but traditionally rely on a common underlying premise that awareness depends on the brain creating a representation that it is presently in a particular mental state (Lau and Rosenthal, 2011). For example, a visual stimulus is initially represented non-consciously in the visual cortices as a first-order representation (the nature of this representation as a brain state is ill-defined by Rosenthal). Higher-order cortical regions (prefrontal and parietal cortices), by re-representing the first-order state, are considered to instantiate conscious awareness of the first-order state. The second-order state remains non-conscious while the first-order sensory representation becomes conscious. Higher-order theories are built on the premise that a “state is conscious only if one is subjectively aware of oneself being in that state” (Rosenthal, 2011). By “one” or “oneself”, Rosenthal refers to a human subject who is experiencing a particular state. Given that Rosenthal equates “conscious awareness” with either “subjective awareness,” “consciousness,” or “awareness” (Lau and Rosenthal, 2011), the premise can be re-written as a “state is conscious only if one is aware of being in that state.” Rosenthal later re-phrases it as “conscious mental states are states we are in some way aware of” (Rosenthal, 2012). Rosenthal stipulates that higher-order theories depend on higher-order awareness. This premise stipulates the necessary condition that one cannot be in a conscious state unless one is aware of it. The second-order representation is a state of awareness of the first-order representation. Using the example of the somatosensory system, it follows that to be in pain one must know or become aware that one is in that mental state. A first-order representation of a noxious stimulus is not considered, by itself, to be subjectively experienced. There needs to be some further higher-order representation (i.e., awareness) of the first-order representation before pain is experienced. Rosenthal also stipulates that the awareness of the mental state is distinct from the qualitative properties of that mental state (e.g., sharp versus a dull throbbing pain). It is the first-order state that is qualitative, not the second-order representation.

Rosenthal (2012) claims that “mental states” (or what he later calls “psychological states”; Rosenthal, 2020) can be either conscious or not conscious. He clearly states that “if someone thinks, desires or feels something but is wholly unaware of doing so, then that thought, desire or feeling is not a conscious state” (Rosenthal, 2012). But how can a mental state such as pain be non-conscious? What Rosenthal seems to be saying is that there are two types of awareness: non-conscious and conscious. He believes that first-order awareness is non-conscious and initially defines the phenomenal properties (i.e., the feeling) of conscious higher-order awareness. The difference between non-conscious and conscious awareness is that the latter is a re-representation of the former. Rosenthal (2012) proposes that this re-representation generates awareness and confers consciousness on first-order non-conscious awareness. To be subsequently aware of second-order awareness requires a higher third-order awareness (or introspective reflection; Rosenthal, 2002). He contends that neither introspective reflection on behaviour nor subjective inference about a feeling state can generate subjective experience. Rosenthal proposes the *higher-order thought theory* that rests on the premise that second-order awareness is a “thought” (Rosenthal, 2002). While the meaning of the concept of “thought” is ambiguous, Rosenthal contends that it must be non-inferential. He claims that subjective experience is a thought that one is in a particular mental state. That thought is a direct (or “immediate”; Rosenthal, 2002) thought (i.e., generated reflexively) and is reported as, for example, “I am in pain,” rather than an indirect thought such as “I think I am in pain” (which would be a third-order awareness and, hence, not given to indicate subjective sensory experience necessarily). Direct thoughts are treated as though they are true according to the subject, but they may not necessarily be true (e.g., when reporting the perceived colour of an object which may, because of contextual presentation, not be the true colour). To be clear, Rosenthal admits that his theory does not address what a thought is (Rosenthal, 2021).

We are sympathetic to certain aspects of Rosenthal’s higher-order thought theory (see section “Applying First Principles to Understand the Neural Basis of Subjective Experience”) but question why a second-order re-representation (i.e., a “thought”) should necessarily lead to subjective experience. Rosenthal’s higher-order thought theory is paradoxical in the sense that it claims that when a brain state is conscious, it is just so because a thought about that brain state is—by definition—conscious. In general, higher-order theories are dependent on neural processes of re-representation and yet the nature of these processes remains undefined. If the re-representation is not essentially different from the neural processes generating the first-order representation, why should a re-representation then necessarily be conscious? Higher-order theories fall short on defining the neural basis of second-order awareness. The challenge remains to explain how second-order awareness is conscious as well as why this awareness should feel like something rather than nothing.

Despite these shortcomings, a role for re-representations (or meta-representations) in subjective experience has considerable support (LeDoux and Brown, 2017; Brown et al., 2019; Cleeremans et al., 2020; LeDoux and Lau, 2020). LeDoux and Brown have advanced their own variation of higher-order representation (HOR) theories called the HOROR theory (i.e., representation of a HOR) (Brown, 2015; LeDoux and Brown, 2017). This theory proposes that subjective experience does not directly emerge from the initial re-representation (i.e., the HOR) of first-order sensory representations but instead depends on a third-order representation (i.e., a HOR of the underlying HOR). The initial HOR allows the integration of memory into the representation while the subsequent third-order representation (of the second-order, non-conscious HOR) generates the subjective experience. LeDoux and Brown (2017) claim that dual HORs are needed to account for the ability to subjectively experience in the absence of any direct sensory inputs. In this way, subjective experience is not dependent on the first-order representations since it can be realised by inputs other than sensory representations (e.g., memory) that are represented in the first HOR. The second HOR is seen as a representation of oneself as being in a particular state of subjective experience and is not necessarily dependent on having an underlying sensory state, although in most cases they do co-occur (Brown, 2015).

LeDoux (2021) has recently proposed a further modification of HOROR which he refers to as a *multistate hierarchical higher-order view.* The central tenet of this new framework is that various forms of memory are progressively incorporated into multiple layers of re-representations before conscious experience is finally generated. LeDoux and Lau (2020) suggests that the re-representations underpinning subjective experience involve the integration of both sensory input and implicit procedural memories. Implicit procedural memory is described as the “learned relations between dynamic neural profiles and sensory inputs” which we have previously referred to as input-output relationships in a neural processing stream (Key and Brown, 2018; Key et al., 2021). New sensory inputs are always experienced within the context of these pre-established relations (LeDoux and Lau, 2020). Brown et al. (2019) allude to the idea that re-representations are a form of monitoring without further explication (we return to the role of monitoring in section “Applying First Principles to Understand the Neural Basis of Subjective Experience”). LeDoux (2020) simply concludes that “the rerepresentation does something to make this lower-level state conscious” and that “something” remains to be resolved. Consequently, the multistate hierarchical higher-order view seems no better at explaining subjective experience then HOROR.

Cleeremans has introduced another modified framework that captures key aspects of the global neuronal workspace and higher-order theories called the *self-organising metarepresentational account* (SOMA) (Cleeremans et al., 2020). This theory builds on the premise that the human brain learns unconsciously to be conscious i.e., it “learns to redescribe its own activity to itself” (Cleeremans et al., 2020). Like LeDoux and colleagues, Cleeremans et al. (2020) proposes that re-representations of first-order states by higher-order monitoring systems are critical for subjective experience. SOMA requires that the re-representation is performed by an observer network that monitors and creates an internal model of first-order states. This observer network gains intrinsic knowledge about sensory states which, although not necessarily conscious, forms the basis for subjective experience but only after this knowledge is hierarchically re-represented reflexively and made globally available. Although the details about how or why this processing should feel like something is left unanswered by SOMA, we like how this account has at least attempted to provide a computational basis of re-representations. SOMA has similarities to our framework which we now present below.

Another interesting theory of consciousness is the integrated information theory (Tononi et al., 1994, 2016). Its authors sought to address some fundamental questions about the relationship between consciousness and brain structure including: why subjective experience depends on some cortical regions and not other brain regions (e.g., the cerebellum) in humans and whether animal brains with vastly different neuroanatomies to humans can support consciousness? However, rather than begin with understanding brain structure-function relationships, Tononi et al. (1994) instead claimed that subjective experience possesses five essential properties (intrinsic, structure, specific, unitary, and definitive) and that each of these properties are realised by physical substrates (i.e., brain structures). The theory further proposes that the level of consciousness can be quantified in terms of maximal integrated information. The approach adopted by Tononi et al. (1994)—i.e., identifying essential properties of consciousness—fails to begin to explain how subjective experience arises in the first place. Rather, it merely identifies properties that are indicative of subjective experience once it is present (a folk analogy would be like saying that the sun is hot, therefore a property of the sun is radiant energy, however this property fails to explain how the sun generates such energy). The integrated information theory is wanting as a theory of consciousness since it ultimately fails to account for how integrated information should lead to brain activity feeling like something rather than nothing. For more detailed critiques of the integrated information theory the reader is referred elsewhere (Cerullo, 2015; Pautz, 2019; Michel and Lau, 2020; Cooke, 2021; Mallatt, 2021). In the next section, we describe an alternate approach whereby we use first principles to instead identify an underlying cause of subjective experience.

## Applying First Principles to Understand the Neural Basis of Subjective Experience

We start with the principle that subjective experience is dependent upon complex neural processing in animal nervous systems (Smart, 1959; Tononi et al., 1994; Halligan and Oakley, 2021). Neural processing is the flow of neural activity (i.e., information) through specialised neural circuits (i.e., modules) performing neural computations (i.e., functions) necessary for specific behaviours. These modules are causally linked in a network. Complex processing is distinguished from simple processing by the presence of hierarchical layers or levels that can act to regulate processing and/or abstract information (Badre and D’Esposito, 2009; Yee, 2019; Eckstein and Collins, 2020; Gilead et al., 2020). Thus, there are two fundamental organisational properties of complex processing—hierarchy and modularisation—both of which facilitate the spatial and temporal execution of multiple interacting parts. They are critical for many behaviours generated by nervous systems (Badre and Nee, 2018; Holroyd and Verguts, 2021; Sherman and Usrey, 2021) and, by extension, to functions such as subjective experience. And yet, it is not enough just to have functional modules—they need to be interconnected in a causal network to generate appropriate outputs (Tononi et al., 1994; Ito et al., 2020). Taken together, subjective experience depends on the hierarchical organisation of specialised neural circuits executing computations (i.e., functions) that are causally driven by interconnections.

The role of complex neural processing in subjective experience is well supported by both experimental interventions and pathological insults in the human nervous system (Key and Brown, 2018; Key et al., 2021). Our challenge is to characterise the nature of this complex neural processing. It is not our intent here to solve the problem of how a physical neural process can subjectively feel like something (i.e., close the explanatory gap), but instead to provide an alternative way of addressing the question. The adopted approach is bottom-up and involves applying fundamental principles to decipher the minimal neural architecture that is necessary for subjective experience.

The *structure-determines-function* principle declares that the function of any system is dependent on its structure; this principle (as with the principle that subjective experience is dependent on specific neural processes) also frames our approach (Brown and Key, 2020). In biology, structure limits function at multiple levels of abstraction: from molecular levels, where the amino acid sequence of proteins governs protein–protein interactions, to gross tissue levels such as in the brain where synaptic connectivity affects behaviour. Given this structure-determines-function principle, we contend that, at an appropriate level of abstraction, there is an organisational structure of neural circuitry that is necessary for subjective experience. To be clear, this means that animals lacking this critical neural circuitry will be incapable of subjective experience. This means that the entire minimal architecture must be present for subjective experience to be realised. We do not contend that specific evolutionary-conserved neuroanatomical structures must be present for subjective experience. Rather, subjective experience depends instead on the presence of relevant neural modules to execute specific functions. This principle explains how sensory experience that is lost after cortical lesions in humans can sometimes return over time as undamaged cortical regions learn new functions (Herbet et al., 2016).

Doerig et al. (2019) refer to consciousness theories that suggest consciousness depends on causal interactions between brain structures as “causal structural theories.” An example is recurrent processing theory (Lamme, 2006) which proposes that visual consciousness arises because of recurrent feedback from higher visual cortices to primary visual cortex. We agree that causal interactions between structures are necessary for consciousness, but it is instead the functions executed by those structures that are necessary, rather merely just the structural interactions themselves. For us, recurrent feedback would be important if, and only if, that feedback was contributing to neural functions necessary for subjective experience. Our approach is to first identify the function and then to characterise the minimal structural requirements (i.e., neural circuitry) to execute that function.

To allay any claims that subjective experience (or its underlying necessary functions) could be multiply realised by any neural circuit (structure), we emphasise the covenant, “appropriate level of abstraction.” As mentioned, multiple realisation does not necessarily apply at all levels of analysis (Brown and Key, 2021a). For example, animal wings can be formed by many different tissues—think insects, bats, and birds—but at the same time share structural features that are necessary for the aerodynamic lift needed for non-gliding flight. The presence of such structures is widely accepted and used to understand the evolution of animal flight (Dudley and Yanoviak, 2011; Chin and Lentink, 2016). The important concept here is that certain structures remain necessary to fulfil the function. Our challenge is to identify a fundamental structural basis for subjective experience (which we address in section “The Neural Architecture Necessary for Subjective Experience”). Provided this structure is non-trivial and sufficiently discerning, it may serve as a biomarker for the potential of subjective experience in an animal.

How does one begin to identify the necessary neural computations underpinning subjective experience? Traditionally, neuroscientists have sought to characterise the neural correlates of consciousness with the aim of reconstructing the flow of information within the brain. We instead advocate a reverse engineering approach whereby we initially ask which critical neural computations must be executed for a nervous system to be capable of subjective experience before seeking to address the nature of those structures that perform the computations. We start with premise that the most fundamental neural process underlying subjective experience of a sensory stimulus is awareness. As discussed above, higher-order theories of consciousness clearly articulate the importance of awareness. Whether unconscious during deep general anaesthesia, or when consciousness is selectively perturbed by local anaesthesia or a brain injury, a patient that is unaware of a sensory stimulus has no subjective experience (Boly et al., 2013). However, awareness is often misconceived as being only the conscious perception of a sensory stimulus or the conscious self-reflection about feelings. We have previously highlighted that preconscious awareness precedes conscious awareness (Key et al., 2021). In any system (artificial or biological), awareness is characterised by the system knowing/understanding (either implicitly or explicitly; Dienes and Perner, 1999) about the state of its inner workings or processes. Although there are many context-specific definitions of understanding (Grimm, 2021), there is general agreement that systems become aware (at least implicitly or preconsciously) when they can predict/infer relationships between internal information structures (e.g., between the inputs and outputs of a processing pathway). It is important to remember that nervous systems have no direct access to sensory stimuli. Their subjective experience of the world is created entirely through awareness of internal processing of neural signals elicited by sensory stimuli. Consequently, nervous systems can only predict/infer the content and quality of sensory stimuli. It is for this reason that nervous systems are often referred to as “prediction machines” since their actions are based on inferences generated by internal models (Seth, 2020). We contend that humans (or any animal) that lack this ability to predict/infer their inner processing (i.e., preconscious awareness) using internal models cannot then subjectively experience the feel of a sensory stimulus. While preconscious awareness is necessary, it is not sufficient for subjective experience.

**FIGURE 1:**
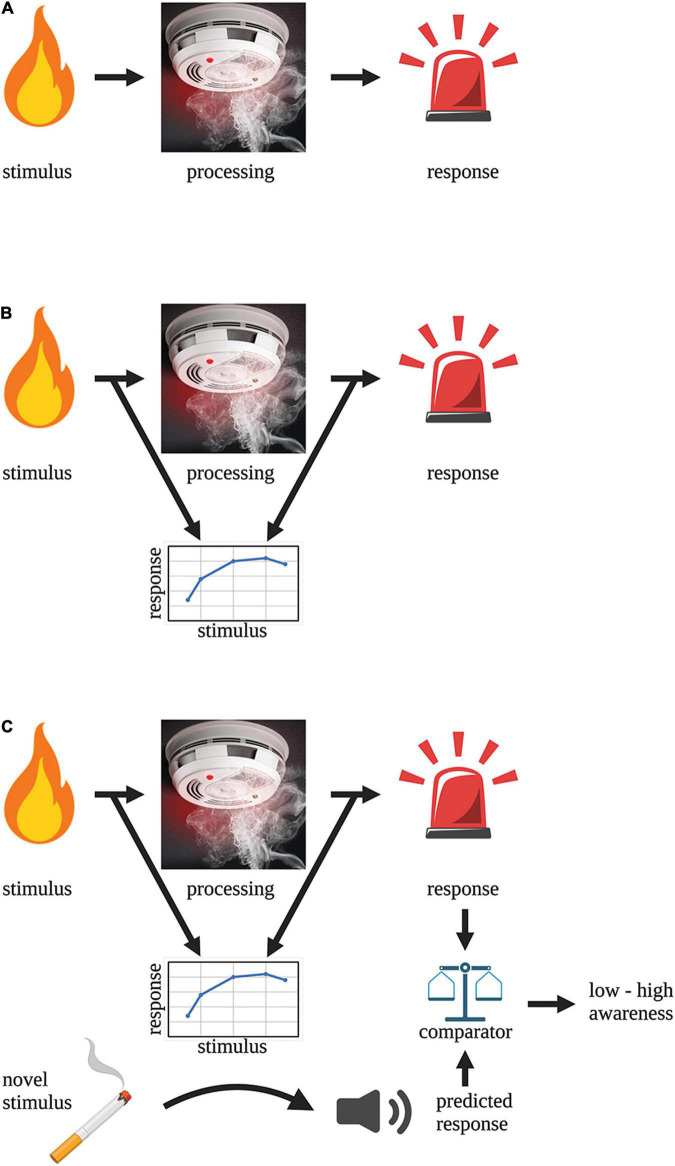
Building “awareness” into a circuit. **(A)** A fire alarm circuit is an example of a simple stimulus-detection (processing)-response circuit that lacks awareness. **(B)** The addition of an external monitoring circuit enables the system to learn the relationship between the stimulus and the response. This monitoring circuit lies external to the processing pathway that is being monitored. **(C)** The addition of a comparator module to the circuit allows the predicted response to be compared to the real response. The accuracy of the prediction is a measure of the “awareness” of the system.

To illustrate how a system could possibly become preconsciously aware about the state of its inner processing we use the toy example of a smoke detector-fire alarm circuit. A smoke detector-fire alarm circuit is a stimulus-response processing pathway (Figure 1A) that—based on the above definition of awareness—clearly lacks preconscious awareness. The stimulus (smoke) detector is embedded within the processing pathway and directly relays electrical signals to the next module to execute a response (alarm). This circuit has no mechanism to model the input-output relationship of its circuit and hence understand what it is processing. To gain preconscious awareness, this system needs an independent second-order circuit to monitor the primary processing pathway (as in hierarchical, complex neural processing as described above). It is not enough that this monitoring circuit simply samples the output of the primary pathway since this provides no new information to the system about what is being processed. The circuit needs to understand the relationship between the input and output of the processing pathway i.e., it must predict/infer what signals (from all possible signals) arising from the stimulus produce an alarm response (Figure 1B). The function of the monitoring circuit is to create a model of the stimulus-response relationship and in doing so, the circuit can predict the response for any given input (Figure 1C). This response prediction can be compared to the real output (via a separate comparator module) and the accuracy of that prediction represents how aware the circuit is about what is being processed. For instance, low accuracy means that the system has poor awareness of what it is processing whereas high accuracy means the system is very aware of what it is processing.

**FIGURE 2:**
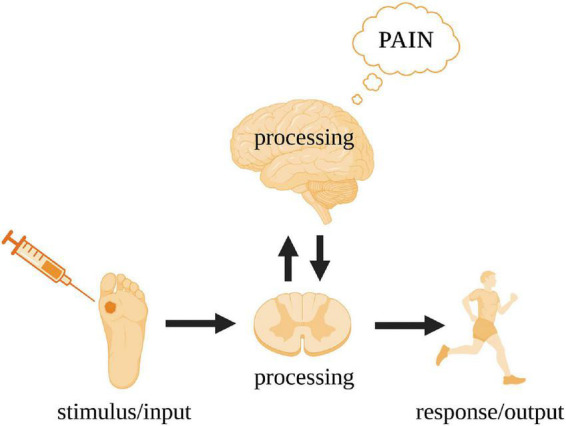
Gross processing pathways underpinning the subjective experience of pain in humans. The spinal cord acts as a conduit for the noxious stimulus input and action output while the cerebral cortex executes the critical neural processing leading to pain (see Figure 3 for details).

We now return to nervous systems and use pain as a case study (Figure 2). In humans, a peripheral noxious stimulus (i.e., input) initially leads to neural processing in the spinal cord and to a motor response (i.e., output). There is no subjective awareness of this processing at the level of the spinal cord—a fact clearly demonstrated by paraplegics (Key and Brown, 2018). Pain arises in higher level circuits that involve reciprocal connections between the spinal cord and the brain. For the brain to be aware of what it is processing, it needs a monitoring device. This monitoring is performed by independent neural circuitry that models the relationship between the input and output of the processing pathway and can predict/infer the nature of the output given any input.

Since nervous system models of awareness can predict outputs for given inputs they are referred to as forward models (McNamee and Wolpert, 2019). While forward models (in the correct architectural framework; see section “The Neural Architecture Necessary for Subjective Experience”) are necessary, they are not sufficient for subjective experience. For instance, forward models fail to account for why awareness should feel like something, rather than nothing. Nonetheless, the neural architecture underpinning the monitoring of internal processes (see section “The Neural Architecture Necessary for Subjective Experience”) is such that it provides a sufficiently discriminatory means of assessing the potential of a nervous system to subjectively experience sensory stimuli.

## The Neural Architecture Necessary for Subjective Experience

**FIGURE 3:**
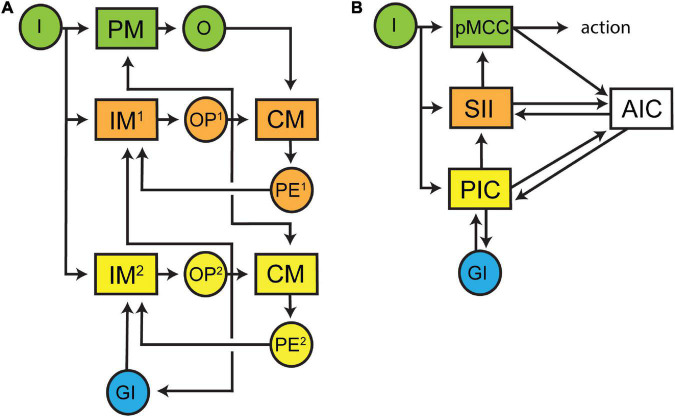
Schematic diagrams of the proposed minimal architectural framework underpinning subjective experience. **(A)** The overall flow of neural processing is represented in three tiers. The sensory processing pathway is coloured green and contains an input (I) into a processing module (PM) and an output (O) from that module. A monitoring circuit (an internal forward model, IM^1^) receives a copy of I and outputs (OP^1^) a prediction of O. OP^1^ and O are compared by a comparator module (CM) and a prediction error (PE^1^) is generated and then fed back to IM^1^ to update its model. A second monitoring circuit (coloured yellow) controls the first monitoring circuit (coloured orange). The second internal forward model (IM^2^) receives input form I and global input (GI) from other processing modules and generates a prediction (OP^2^) of OP^1^. OP^2^ is compared against OP^1^ in a CM and the prediction error (PE^2^) is fed back into IM^2^ to update its model. OP^2^ is also broadcast globally to update other processing modules. **(B)** The framework presented in panel **(A)** can be mapped on to cortical regions processing noxious inputs. Inputs initially enter the posterior mid-cingulate cortex and generate descending motor outputs. These motor outputs are also relayed to the anterior insular cortex where they are compared with predictions arising from tiered forward models located in the somatosensory area II and posterior insular cortices. These predictions hierarchically descend on to the posterior mid-cingulate cortex where they modulate motor outputs. Feedback (prediction errors) from the anterior insular cortex to both the somatosensory II and posterior insular cortices maintain the accuracy of predictions. The posterior insular cortex predictions are further modulated by reciprocal connections with multiple cortical areas processing other sensory inputs.

By understanding that preconscious awareness depends on forward models in monitoring circuits, we can now characterise the neural architecture (modules and connectivity) that is necessary for their function by applying the structure-determines-function principle. The basic structure of a forward model is well described in control theory (Tin and Poon, 2005) and is roughly sketched in Figure 1C and more thoroughly presented in Figure 3 within a framework supporting subjective experience. The architecture is built around a simple processing pathway consisting of an input (I) into a processing module (PM) and an output (O) from that same module (coloured green, Figure 3). The monitoring circuit (coloured orange, Figure 3) resides outside of this processing pathway and consists of a first-order internal forward model (IM^1^) that receives a duplication of the input entering the processing pathway. The output of IM^1^ is a prediction (OP^1^) of the output of the processing pathway (O). Feedback is essential to training IM^1^ and increasing the accuracy of OP^1^. Therefore, OP^1^ is used as input into a comparator module (CM) that also receives a copy of O. The output of the CM is a prediction error (PE^1^) which is the difference between OP^1^ and O. PE^1^ is then fed back into IM^1^ where it is used to adjust internal model parameters. By feeding OP^1^ back into PM, the internal model can bias processing toward the predicted output. In doing so, awareness has gained the physiological functions of noise reduction and decreased processing times. These functions may contribute to any evolutionary advantage of subjective experience (Graziano, 2014).

Using preconscious awareness (OP^1^) of an isolated input-output processing pathway to control the behaviour of an animal is likely to have catastrophic effects unless that awareness accounts for competing sensory inputs as well as other internal processes. To generate an integrated awareness, a second internal forward model (IM^2^) needs to control IM^1^ (coloured yellow, Figure 3). A second internal model is favoured here since internal models are necessary for optimal processing control (Conant and Ashby, 1970; Francis and Wonham, 1976; Tin and Poon, 2005; Huang et al., 2018; McNamee and Wolpert, 2019; Madhav and Cowan, 2020). This stacking of internal models is also consistent with the hierarchical design features of nervous systems. IM^2^ receives inputs from I as well as multiple global inputs (GI, coloured blue, Figure 1). The output of IM^2^ (OP^2^) is passed to the comparator together with OP^1^ and the prediction error (PE^2^) is then used to update IM^2^. OP^2^ is fed back into IM^1^ where it can bias processing toward OP^2^. OP^2^ can also be globally broadcast to modulate many different internal processes (which would be consistent with the global neuronal workspace theory). This second internal model may explain how it is possible to subjectively experience in the absence of the sensory stimulus as proposed by LeDoux and Brown (2017) in their HOROR model with tiered re-representations.

We have previously mapped the various modules in Figure 3A to neuroanatomical structures in the human brain (Figure 3B) in relation to the subjective experience of pain (Key and Brown, 2018). In brief, sensory inputs enter the posterior middle cerebral cortex (PM), somatosensory cortex II (IM^1^) and posterior insular cortex (IM^2^). Each of these areas project to the anterior insular cortex (CM). The posterior insular cortex also broadly connects with multiple cortical regions (GI). These structures fulfil four important conditions of the circuitry in humans (see Key and Brown (2018)). First, the anatomical interconnectivity of these regions is consistent with the proposed architecture. Second, neurophysiological recordings have revealed that the temporal activation of these regions matches their predicted sequence of firing within this hierarchy. Third, lesions and direct electrical stimulation of these cortical regions produces sensory deficits. Fourth, each of these regions have been shown to appropriately generate either predictions, comparisons, or prediction errors.

It should be clear now that both the stacked internal models and their networked connectivity, as we have described here, are consistent with our original proposed features of complex neural processing—hierarchical organisation, modularisation, and causal interconnectivity. These stacked forward models provide both rapid and selective control of the processing pathway at a local level as well as enable integrated control necessary for global homeostasis. We refer to this neural architecture as the “*hierarchical forward models algorithm*” and it is consistent with the known anatomical substrates underpinning the neurophysiological processing of noxious stimuli in the human brain (Key and Brown, 2018). We postulate that this neural architecture (or slightly modified versions of it) is necessary for an animal to subjectively experience any sensory stimulus. (We make no claims to its being a sufficient condition). While our framework does not prescribe the fine structure of the processing modules, it demands that these modules can execute the appropriate computations to generate the needed output functions (i.e., predictions, comparison, and prediction errors) in an appropriate temporal order.

## Conclusion

Our strategy applies two basic principles (first, subjective experience is contingent on neural processing executing specific neural functions; and second, structure-determines-function) to define a minimal neural architecture necessary for subjective experience. Since this approach was never intended to bridge the gap between preconscious and conscious awareness, it has allowed us to avoid the contentious and more challenging question of why subjective experience should feel like something rather than nothing. For now, this question remains unanswered. Nonetheless, our framework has already provided insights into the sorts of organisms that most likely lack subjective experience, such as plants (Brown and Key, 2021b), insects (Key et al., 2021), molluscs (Key and Brown, 2018), and fish (Brown and Key, 2021a). It should be noted that it is not forward models *per se*, but rather it is their deployment and implementation within the correct architectural framework—as revealed by the *hierarchical forward models algorithm*—that countenances the likelihood of subjective experience. For example, while processing of noxious stimuli in drosophila involves hierarchical processing modules that act in parallel, the underlying circuitry lacks the necessary interconnectivity required to execute the computations (predictions and predictions errors) of either first-order or second-order forward models (Key et al., 2021).

While our *hierarchical forward models algorithm* shares some similarities with other higher-order theories of consciousness, it has enabled a major advance by allowing identification of some necessary neural computations and the requirement of specific neural architectures for their execution. Together, these criteria constrain the types of nervous systems that can give rise to subjective experience. Equating preconscious awareness with internal models and their central importance in control of neural processing has also provided new insights into possible functional advantages of subjective experience.

Another popular approach in perceptual processing is predictive coding (Friston, 2010). While predictive processing advocates for hierarchical internal models, there are some major differences with our *hierarchical forward models algorithm.* The models in predictive processing reside within the processing pathway. As such, these models are designed to predict the causes of sensory stimuli and to explain content of what is experienced rather than the awareness of that content. In our algorithm, the forward models predict the outcome of processing and hence provide external awareness of content. Importantly, both approaches adopt the structure-determines-function principle and claim that the execution of the internal models demands defined neural architectures. It is not incidental that the evolution of neural architectures supporting hierarchical internal models has clearly been instrumental for both perceptual processing and for subjective experience.

## Data Availability Statement

The original contributions presented in the study are included in the article/supplementary material, further inquiries can be directed to the corresponding author/s.

## Author Contributions

All authors contributed to the research and writing of this manuscript.

## Conflict of Interest

The authors declare that the research was conducted in the absence of any commercial or financial relationships that could be construed as a potential conflict of interest.

## Publisher’s Note

All claims expressed in this article are solely those of the authors and do not necessarily represent those of their affiliated organizations, or those of the publisher, the editors and the reviewers. Any product that may be evaluated in this article, or claim that may be made by its manufacturer, is not guaranteed or endorsed by the publisher.
